# Social support and older adult falls

**DOI:** 10.1186/s40621-016-0070-y

**Published:** 2016-02-08

**Authors:** Laura Durbin, Rebekah J. Kharrazi, Rebecca Graber, Thelma J. Mielenz

**Affiliations:** 1 Department of Epidemiology, Columbia University Mailman School of Public Health, 722 West 168th St., Room 516, New York, NY 10032 USA; 2 Department of Social Sciences, University of Alabama at Birmingham, 1720 2nd Ave. S, Birmingham, AL 35233 USA

**Keywords:** Aging, Accidental falls, Social support, Risk factors

## Abstract

**Background:**

Social support has been shown to be associated with various positive health outcomes among older adults but has not been previously examined in relation to falls, which are a serious health concern among older adults.

**Findings:**

This study (*n* = 1000) uses multivariable logistic regression to evaluate the relationship between social contact and perceived availability of social support and falls among older adults. When adjusting for demographic and other covariates neither relationship was significant.

**Conclusions:**

This study does not find evidence to suggest that social support could be a prevention measure for falls. Future research on this topic should focus on careful definition and precise measurement of the social support construct.

## Objective

Falls, which are frequent among older adults, are a serious concern for this population as they often cause consequential injuries and can even be fatal (WHO 2007). Nearly 1 in 3 older adults experience a fall each year, and half of these individuals fall more than once (CDC 2015). Although social support (including perceived support) has been found to be a protective factor for depression, anxiety, and physical disability among older adults, it has not previously been examined explicitly in relation to fall events (Cole and Dendukuri 2003; Vink et al. 2008; Taylor 2011).

Social support has been defined as the perception or experience that a person is cared for, valued, and esteemed by others, and is a member of a social network that provides mutual assistance (Wills 1991). Barrera (1986) suggested in a review of the literature that social support can take the following forms: social embeddedness, perceived support, and enacted support. Since then, other constructs have become accepted in the social support field (such as instrumental, emotional, and informational support), with perceived social support continuing to be recognized as a valuable construct (Langford et al. 1997; Taylor 2011).

It has been suggested that social support could act as a protective factor against falls, perhaps by encouraging older adults to be more attentive to hazards in the environment or by ensuring that at-risk individuals receive help for completing risky tasks, such as reaching for far away objects or taking out the garbage (Faulkner et al. 2003; Hosseini and Hosseini 2008). This study evaluates the relationship between social support and falls among a community-dwelling population of older adults.

## Methods

This cross-sectional study is a secondary analysis of the New York City Housing Authority (NYCHA) Senior Survey, which surveyed NYCHA building residents aged 65 and older via telephone interviews in June 2009. Participants were randomly selected from the more than 65,100 residents meeting the age criteria, with a final sample size of 1,036, which exceeded the prestudy sample size calculation of 142 (80 % power, 5 % alpha, medium anticipated effect size). NYCHA’s published findings contain further details regarding study measures and questions (Parton et al. 2011). This secondary analysis study was approved by Columbia University’s Institutional Review Board.

The outcome of interest was a dichotomous measure of self-reported falls during the past year. The primary predictors of interest were social contact and perceived availability of social support. Social contact is a component of social embeddedness, which is in turn a necessary antecedent of social support (Barrera 1986; Langford et al. 1997). In this study, social contact was measured by the following survey question: “During the past week, did you talk with relatives, friends, or neighbors on the telephone?” Perceived availability of social support attempts to determine an individual’s confidence that appropriate support would be available to them if they needed it (Barrera 1986). In this study this construct was measured with the following question: “Is there a friend, relative, or neighbor who could assist you for a few days if necessary?”.

Bivariable analyses were conducted between falls and both predictors, as well as between falls and each of the covariates, using the Chi-squared test. Two logistic regression models, one for each social support construct, were used to conduct multivariable analyses. Covariates, selected among available measures that have previously been shown to be associated with falls among older adults, are reported in Table 1. All covariates were initially included in the logistic regression models. The covariates that were nonsignificant at the bivariate level were removed first, followed by the significant variables. The change-in-estimate method with a 10 % change criterion was used to remove covariates (Walter and Tiemeier 2009).

**Table 1:** Characteristics of fallers and non fallers, NYCHA residents, New York City, 2009

	Fallers (*n* = 280)	Non Fallers (*n* = 720)
	N (%)	N (%)
Social Support (*n* = 1000)		
Perceived availability	195 (69.64)	526 (73.06)
Social contact	251 (89.64)	647 (89.86)
Age (*n* = 1000)		
65-74	161 (57.50)	401 (55.69)
75-84	95 (33.93)	264 (36.67)
85+	24 (8.57)	55 (7.64)
Ethnicity/Race^a^ (*n* = 1000)		
Black	144 (51.43)	430 (59.72)
White	33 (11.79)	62 (8.61)
Hispanic	95 (33.93)	193 (26.81)
Asian/Pacific Islander	8 (2.86)	35 (4.86)
Gender (*n* = 1000)		
Male	75 (26.79)	169 (23.47)
Female	205 (73.21)	551 (76.53)
Type of Household (*n* = 1000)		
Single	163 (58.21)	380 (52.78)
Multiple	117 (41.79)	340 (47.22)
Type of Development (*n* = 1000)		
Family	226 (80.71)	585 (81.25)
Mixed	13 (4.64)	42 (5.83)
Senior Only	41 (14.64)	93 (12.92)
Vision Difficulties^a^ (*n* = 975^b^)		
No Difficulty	49 (18.35)	218 (30.79)
Low Difficulty	137 (51.31)	338 (47.74)
High Difficulty	81 (30.34)	152 (21.47)
Self-Described Health^a^ (*n* = 994^b^)		
Good	69 (24.73)	328 (45.87)
Not Good	210 (75.27)	387 (54.13)
Presence of Health Issue		
Dizziness^a^ (*n* = 990^b^)	172 (62.32)	218 (30.53)
Stroke^a^ (*n* = 993^b^)	32 (11.51)	28 (3.92)
Heart Attack^a^ (*n* = 995^b^)	37 (13.26)	36 (5.03)
Diabetes^a^ (*n* = 995^b^)	135 (48.39)	229 (31.98)
Osteoporosis^a^ (*n* = 978^b^)	86 (32.09)	171 (24.08)
Arthritis^a^ (*n* = 988^b^)	211 (77.01)	404 (56.58)
Depression^a^ (*n* = 988^b^)	88 (32.35)	77 (10.75)
Physical Activity^a^ (n = 996^b^)		
Active	175 (62.72)	542 (75.59)
Inactive	104 (37.28)	175 (24.41)

*NYCHA* New York City Housing Authority

^a^Chi-square values significant at *p* < .05

^b^N values smaller than 1000 reflect missing data for specific covariates

## Findings

The overall response rate for the NYCHA study was 34.7 %, with a cooperation rate of 93.4 % for individuals reached by phone. Thirty-six individuals were excluded for missing data on falls or social support constructs, resulting in a study population of 1,000. The mean age was 74, with a maximum age of 95. Participants represented several races (57.4 % black, 28.8 % Hispanic, 9.5 % white, 4.3 % Asian/Pacific Islander) and were mostly female (75.6 %). A large percentage of participants reported social contact (89.8 %) and a perceived availability of social support (72.1 %). Nearly one in three participants (28 %) reported falling in the past year. A comparison between fallers and non-fallers is outlined in Table 1. In bivariate analyses, neither social contact nor perceived availability of social support was significantly associated with falls.

Variables adjusted for in both final social support models included age, gender, race/ethnicity, vision difficulties, self-described health, dizziness, osteoporosis, arthritis, stroke, heart attack, diabetes, depression, and physical activity. Table 2 presents only the point estimates for the social support exposure. In both final models, the variables that were significant include: gender, self-described health, dizziness, arthritis, stroke, diabetes, depression, and physical activity. Gender was not originally significant in bivariate analyses, but was significant in the final model. Age, ethnicity/race, vision difficulties, heart attack, and osteoporosis were not significant covariates. Without adjustment, both perceived availability of social support (OR = 0.846, 95 % CI: 0.620,1.146) and social contact (OR = 0.976, 95 % CI: 0.620,1.538) had odds ratios below 1.00, with no indication of significant effects. When adjusting for the covariates, perceived availability of social support still reported an odds ratio below 1.00 (OR = 0.948, 95 % CI: 0.650,1.384), while the odds ratio for social contact changed direction to above 1.00 (OR = 1.320, 95 % CI: 0.746,2.338), though neither of these adjusted effects was significant (Table 2).

**Table 2:** Logistic regression models predicting fall events among NYCHA residents, New York City, 2009

Primary predictor (two models)	Unadjusted OR (95 % confidence Interval) (*n* = 1000)	Adjusted OR (95 % confidence Interval) (*n* = 912^b^)
Social Contact	0.976 (0.620-1.538)	1.320^a^ (0.746-2.338)
Perceived availability of social support	0.846 (0.625-1.146)	0.948^a^ (0.650-1.384)

*NYCHA* New York City Housing Authority

^a^Adjusted for age, self-described health, race/ethnicity, gender, dizziness, vision difficulties, arthritis, osteoporosis, heart attack, stroke, diabetes, depression, and physical activity

^b^An n value smaller than 1000 reflects missing data for the covariates that were used during adjustment

## Discussion

This study found no significant association between perceived availability of social support or social contact and falls among older adults. The null finding indicates that there is not currently evidence to support focusing on social support as a prevention measure for falls. It is worth noting, however, that in the unadjusted model, social contact originally had an odds ratio below 1.00, but the directionality of the finding changed to an odds ratio above 1.00 after covariates were added to the model. It is possible that substantial confounding occurred, but the close proximity of the odds ratio to 1.00 suggests that directionality may have changed simply due to chance. Perceived availability of social support had an odds ratio below 1.00 both before and after adjustment.

In addition to being one of the first studies to examine social support and its relationship to fall events among older adults, a primary strength of this study is its population. Studies of falls among older adults have tended to focus on sociodemographically homogenous, and particularly white, populations (Chan and Fong 2013; Choi et al. 2014). Other study strengths include a large sample size and nearly complete reporting for most variables.

A major limitation of this study is the cross-sectional design, which is unable to establish a temporal order between fall events and the social support constructs. The timing mentioned in the questions is an important limitation as well. The falls question asks if individuals fell during the past 12 months, whereas the social contact variable references the “past week” and the perceived availability of social support variable asks individuals to reference their perceptions about what could happen in the future, presumably based in part on their past and recent experiences. For the purposes of this analysis, we have treated these social support variables as relatively constant variables without variation over time. However, it is very possible that after experiencing a major event, such as a fall, that an individual’s social contact and perceived availability of social support could change dramatically, with relatives, neighbors, and others close to the individual providing more social support since the event. Individuals who were receiving limited social contact and support before a fall, may have reported receiving much more contact and support at the time of the survey’s administration.

Further limitations include self-reported data and the use of a dichotomous falls variables, which does not provide as much information as would the exact number of fall events experienced (Parton et al. 2011). Questions that were used to determine social contact and perceived availability of social support were not originally written to measure these specific social support subdomains. This lack of precision in measuring each subdomain could be the cause of the null findings. Additionally, the dataset utilized did not include many other factors that, when combined with social support, could have a positive impact on falls (medications and fall risk assessed by physician, home assessments, etc.).

Future studies should thus consider using longitudinal designs, monthly falls calendars and the National Institutes of Health (NIH) Patient Reported Outcomes Measurement Information System (PROMIS) measures, which currently cover seven subdomains of social support precisely, as they were constructed using modern measurement theory (Hannan et al. 2010). It is possible that when operationalized in a more appropriate way, social support will indeed be found to be associated with falls. However, it is also possible that social support does not hold any relevance for falls among older adults.

## Abbreviations

NIH: National Institutes of Health
NYCHA: New York City Housing Authority
PROMIS: Patient Reported Outcomes Measurement Information System
